# What is the Best Configuration of Wearable Sensors to Measure Spatiotemporal Gait Parameters in Children with Cerebral Palsy?

**DOI:** 10.3390/s18020394

**Published:** 2018-01-30

**Authors:** Lena Carcreff, Corinna N. Gerber, Anisoara Paraschiv-Ionescu, Geraldo De Coulon, Christopher J. Newman, Stéphane Armand, Kamiar Aminian

**Affiliations:** 1 Laboratory of Kinesiology Willy Taillard, Geneva University Hospitals and University of Geneva, 1205 Geneva, Switzerland; stephane.armand@hcuge.ch; 2 Laboratory of Movement Analysis and Measurement, Ecole Polytechnique Fédérale de Lausanne, 1015 Lausanne, Switzerland; anisoara.ionescu@epfl.ch (A.P.-I.), kamiar.aminian@epfl.ch (K.A.); 3 Pediatric Neurology and Neurorehabilitation Unit, Department of Pediatrics, Lausanne University Hospital, 1011 Lausanne, Switzerland; corinna.gerber@chuv.ch (C.N.G.); christopher.newman@chuv.ch (C.J.N.); 4 Pediatric orthopedics, Geneva University Hospitals, 1205 Geneva, Switzerland; geraldo.decoulon@hcuge.ch

**Keywords:** cerebral palsy, gait, inertial sensors, gait events, spatiotemporal parameters

## Abstract

Wearable inertial devices have recently been used to evaluate spatiotemporal parameters of gait in daily life situations. Given the heterogeneity of gait patterns in children with cerebral palsy (CP), the sensor placement and analysis algorithm may influence the validity of the results. This study aimed at comparing the spatiotemporal measurement performances of three wearable configurations defined by different sensor positioning on the lower limbs: (1) shanks and thighs, (2) shanks, and (3) feet. The three configurations were selected based on their potential to be used in daily life for children with CP and typically developing (TD) controls. For each configuration, dedicated gait analysis algorithms were used to detect gait events and compute spatiotemporal parameters. Fifteen children with CP and 11 TD controls were included. Accuracy, precision, and agreement of the three configurations were determined in comparison with an optoelectronic system as a reference. The three configurations were comparable for the evaluation of TD children and children with a low level of disability (CP-GMFCS I) whereas the shank-and-thigh-based configuration was more robust regarding children with a higher level of disability (CP-GMFCS II–III).

## 1. Introduction

Cerebral palsy (CP) is the most frequent motor disorder in children with a prevalence of 1.8:1000 births in Europe [1]. For the majority of children with CP who achieve community ambulation, an objective evaluation of their gait is necessary to accurately identify and understand gait impairments, in order to provide adequate and efficient treatment [2]. Such assessments are usually performed in laboratory settings, during standardized clinical gait analysis (CGA). Numerous gait parameters including spatiotemporal parameters (STP), kinematics, kinetics, and muscle activity can be quantified using an optoelectronic (3D motion capture) system, force plates, and an electromyography system [3].

During gait, foot strikes (FS) and foot offs (FO) succeed each other and constitute the base for gait cycle segmentation and the computation of STP. Gait events can be computed from a single force plate, restricting the measure to one step at a time. With the combination of several force plates and an optoelectronic system, it is possible to determine these events over several steps [4]. The advantage of this method is to calculate spatial parameters, such as the stride length, directly from the marker positions. Despite its wide use in CGA [5,6,7,8], the optoelectronic system has some unavoidable limitations. It suffers from inaccuracies linked to instrumental errors, soft tissue artefacts (the relative displacement between the markers and the underlying bone), and marker misplacement due to anatomical landmark palpation difficulties [8]. Since STP estimation is mainly based on the tracking of markers located on the heels and toes, soft tissue artefacts and marker misplacement are not likely to induce major errors. Therefore, instrumental errors are the main sources of inaccuracy in this situation. In 2005, a review reported mean errors for marker distance estimates between 0.1 mm and 5.3 mm depending on the systems [9]. Thanks to the improvement in camera resolution, these errors were expected to decrease. Recently, Di Marco et al. reported that the number of cameras, the calibration volume, and the calibration procedure can induce errors in the marker trajectories reconstruction between 0.2 mm and 1.7 mm [8,10]. Moreover, the optoelectronic system restrains the measurement volume to the laboratory and thus may hinder the patient’s natural gait. As a result, gait parameters obtained through CGA are not fully representative of the usual and daily walking habits (described as ‘performance’ [11]) of children with CP [12,13,14]. Performance can currently be estimated through questionnaires and clinical observations which have the inherent drawback of being subjective and evaluator-dependent, and thus associated with potential bias [15,16]. Therefore, there is a need to objectively assess gait performance in daily-life conditions in order to complement CGA, thus enhancing therapeutic choices for children with CP based on real-life data. Wearable inertial sensors can be used to fulfill this need.

Inertial sensors are microelectromechanical systems, including accelerometers and gyroscopes, contained in small casings that can be carried by the patient without restrictions for several hours of measurements. A wearable sensor-based gait analysis system relies on a sensor configuration and an associated algorithm. Many sensor configurations have been tested for STP estimation varying in numbers of sensors (single or multiple), type (single or triaxial), and location on the body [17]. As, in human gait, most body motion comes from the lower limbs, sensors are commonly fixed on the lower limbs [17]. Gait events are detected according to specific features appearing on accelerometer and gyroscope signals in the time and frequency domains [18,19,20]. Spatial parameters are computed from these signals through methods dependent on the sensor location [17,20]. Yang et al. defined three categories of algorithms: the abstraction models where the spatial parameter is estimated from a black box model building the relationship between the sensor measurements and the output (e.g., artificial neural networks, third-order polynomial model, etc.); the human gait models which use the geometric properties of the lower limbs to estimate stride length; and the direct integrations which consist of integrating the acceleration in the global frame between two specific points of the gait cycle in order to calculate stride velocity (simple integration) and stride length (double integration).

The validity of gait event detection and STP computation from wearable devices has been studied in healthy adults, adults with disease, or elderly population, but scarcely in children [21,22,23,24]. Lanovaz et al. found that, in healthy children, a system of six inertial sensors was valid for temporal detection but showed consistent bias for spatial parameters estimation with the gait model method [25]. In children with CP, a protocol named “Outwalk” has been developed to measure trunk and lower-limb 3D kinematics using an inertial and magnetic measurement system, but the authors did not assess STP [26,27]. Laudanski et al. observed that gait analysis based on sensors on the feet bring more error than shank-mounted sensors, especially for abnormal gait pattern such as toe-out walking [28]. In children with CP with low to mild gait impairments, Bregou-Bourgeois et al. used two foot-worn inertial sensors and the direct integration method to estimate STP and showed good accuracy, precision, and agreement against an optoelectronic system for the estimation of stride length, walking speed, and foot angles with regard to the ground [29]. These previous studies pointed out the difficulty of assessing gait in children with CP with inertial sensors since these children cannot achieve the expected movement properly (i.e., full foot contact during gait [29], good alignment in static, or pure flexion/extension of the knee for functional calibration of the sensor axes [26]). Considering these challenges and since gait patterns are very heterogeneous among patients with CP who are able to walk [2], the usability of other sensor set-ups deserves exploration in order to determine which is the most appropriate one. Furthermore, children with a higher level of impairment should be considered as they may benefit even more from the assessment of their gait performance.

The aim of this study was to evaluate and compare the measurement performance of three wearable configurations and to identify the most appropriate set-up for gait assessment of children with CP. For this purpose, a standard optoelectronic system was used as a reference to evaluate event detection and STP computation.

## 2. Materials and Methods

This study was an observational case-control validation study with a single center setup.

### 2.1. Participants

Fifteen children and adolescents with CP and eleven age- and sex-matched typically developing (TD) controls were evaluated in the laboratory of kinesiology within Geneva University Hospitals (HUG). Participants of the CP group were recruited from the patients followed at the HUG pediatric orthopedics unit if they met the following inclusion criteria: (a) aged between 8 and 20 years; (b) diagnosis of CP; (c) ability to walk in the community with or without mechanical walking aids; and (d) with a level of Gross Motor Function Classification System (GMFCS) [30] between I and III. For the control group, children were recruited among collaborators’ or patients’ acquaintances. The exclusion criteria for both groups were those that precluded adequate participation in the measurement sessions (mental age <8 years, severe visual disorder, attention deficit, and other significant behavioral issues). All participants provided written consent, and the protocol was approved by the hospital’s institutional ethical committee (CCER-15-176).

### 2.2. Protocol

**Figure 1:**
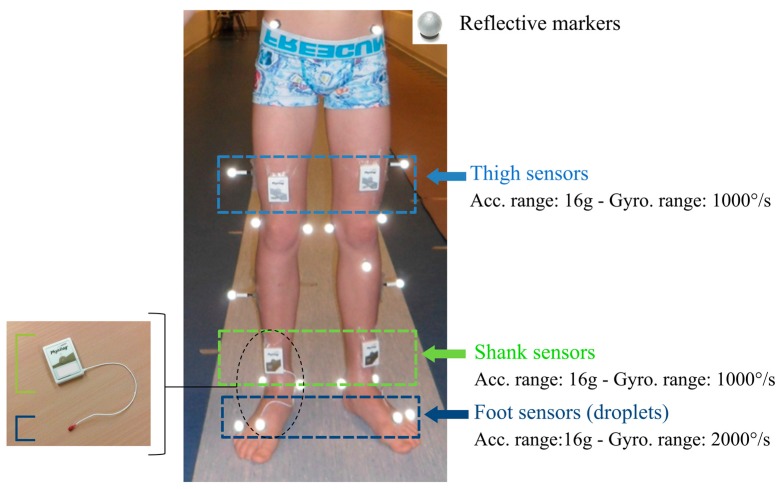
Equipment on the participant’s lower limbs: reflective markers for the optoelectronic system and inertial Physilog sensors for the wearable configurations on the thighs, shanks, and feet. Feet sensors are embedded in a droplet (red PCB resin on the picture on the left), corresponding to deported 6D inertial units connected to the shank sensors.

A trained experimenter measured the following anthropometric data: leg, thigh, shank, and foot lengths, as well as knee, ankle, and pelvis widths which were required inputs for the computation of gait parameters (by the optoelectronic and the wearable systems). Participants were subsequently equipped with the equipment described below, as illustrated in Figure 1 (all sensor configurations were worn at the same time), and asked to walk barefoot on a 10-m walkway. They walked at their self-selected speed for six to eight trials, in order to record a sufficient quantity of gait cycles per participant (minimum of 60 cycles). Participants with a high level of disability (GMFCS III) were allowed to perform the evaluation with their mechanical aid (e.g., walker).

### 2.3. Reference Spatiotemporal Parameters of Gait Using Laboratory Setting

#### 2.3.1. Equipment

A set of 35 reflective markers (14 mm diameter) was placed on specific anatomical landmarks of the participant’s head, trunk, pelvis, arms, thighs, shanks, and feet according to the full-body Plug-In-Gait model (Davis, 1991) (Figure 1). Leg lengths, pelvis, knee, and ankle widths previously measured were used as inputs in the Plug-In-Gait model. Marker trajectories were recorded by a twelve-camera optoelectronic system (Qualisys Motion capture systems, Göteborg, Sweden) set at a sampling frequency of 100 Hz. Two force plates (AMTI Accugait, Watertown, NY, USA) embedded in the middle of the walkway recorded ground reaction forces at 1000 Hz.

#### 2.3.2. Spatiotemporal Parameters Computation

As it is the clinical standard for in-laboratory gait analysis, the force plates and optoelectronic systems were set as reference to compare the three wearable configurations [31,32]. When an entire foot contact on the force plate was available, the gait events (FS and FO) were determined from the vertical ground reaction force based on a threshold sets to 20 N. The subsequent occurrences of the same events were detected on the marker trajectories by the kinematic-based model [33]. All rated events were manually checked frame by frame on the sagittal view of the heel and toe markers in the MOKKA software [34]. The stride time was calculated as the time difference between two successive FS, the stride length was computed as the distance separating the heel markers at two successive FS times, and the stride velocity was calculated as the stride length divided by the stride time for each gait cycle [35].

### 2.4. Spatiotemporal Parameters of Gait Using Wearable Setting 

#### 2.4.1. Equipment

Six synchronized inertial sensors (Physilog4^®^, Gait Up, Renens, Switzerland) were used in this study. Physilog4^®^ is a standalone device (dimensions: 50 mm × 37 mm × 9.2 mm, weight: 19 g) comprising a triaxial accelerometer, triaxial gyroscope, triaxial magnetometer, and barometer with adjustable ranges, battery, a memory unit, and microcontroller. The magnetometer and barometer were disabled. The sampling frequency was set at 100 Hz. Sensors were fixed on the participant’s thighs, shanks, and feet bilaterally using a hypoallergenic adhesive film (Opsite Flexigrid, Smith & Nephew Medical, Hull, UK), as shown in Figure 1. Sensors on the feet were embedded in a droplet (a small deported 6D sensor unit containing the 3D accelerometer and 3D gyroscope) in order to be appropriate for measuring activities in daily life settings: either barefoot or with shoes. Shank and thigh sensors were always oriented in the same way relating to the thigh and the shank, as shown in Figure 1.

The optoelectronic and wearable systems were synchronized using an additional Physilog4^®^ receiving a trigger signal from one camera for every trial start and stop. The synchronization was performed in post-treatment according to the pulse train recorded by the additional sensor.

#### 2.4.2. Sensor Configuration and Associated Algorithm

Three configurations were chosen, which all have the following in common: (1) algorithms described in research studies with high number of subject (*n* > 800) [36,37,38], (2) commercially available sensors, (3) easy to setup sensors and possibility of long-term measurements in daily-life, and (4) validation against laboratory system in adult populations (healthy or with disease). The three configurations use different sensor locations: first, the ‘Shanks and Thighs’ (ShTh) with four sensors fixed on both thighs and shanks; second, the ‘Shanks’ (Sh) with only one sensor on each shank; and finally, the ‘Feet’ with one sensor on each foot. The algorithms associated to the three above-mentioned configurations were used in this study: the double pendulum gait model in ShTh [39] and Sh, with a reduced number of sensors in Sh [40], and the direct integration method in Feet [41,42]. The ShTh and Sh methods have the same algorithm for gait event detection. Table 1 provides details about the three configurations regarding sensor location, algorithms, event detection, output gait parameters, and information about validation of the algorithms.

### 2.5. Data Analysis

Two gait events, FS and FO, as well as three STP, stride time, stride length, and stride velocity, were extracted from the three wearable configurations for each participant and each side, and compared cycle by cycle with the same outputs extracted from the reference (optoelectronic system). These parameters were selected as they are fundamental descriptors of gait and the computation of other STP parameters is based on them.

For TD participants, the parameters extracted from left and right sides were congregated since there was no difference between limbs. For children with CP-GMFCS I, the parameters were separated into paretic and non-paretic limbs according to their clinical profiles. Children with CP-GMFCS II and III were all bilaterally affected and, therefore, left and right sides were congregated and reported as paretic limbs.

The number of non-detected gait cycles, resulting from irregularities in the signal pattern (mostly due to pathological gait), was reported for each method. In cases where gait events were detected but no associated STP was computed, the cycle was not considered and reported as non-detected.

### 2.6. Statistical Analysis

We conducted descriptive analyses to evaluate the consistency of the three configurations with regard to the reference system. To that purpose, error (mean and standard deviation of the difference with the reference) was determined for each configuration. Regarding gait event detection, positive/negative values stand for late/early gait event detection, respectively, and over/underestimation of STP. The analyses were performed for the subgroups of TD, CP-GMFCS I (subdivided into paretic and non-paretic sides), and CP-GMFCS II and III in order to observe the influence of the impairment level on the error.

Spearman’s correlation coefficients (r) were used to evaluate the linear association between each configuration and the reference for the three STP in each subgroup. Furthermore, to quantify the agreements between each configuration and the reference system and visually represent the distribution of the errors, a graphical analysis through Bland–Altman plots was performed for each configuration for the three STP for each group.

## 3. Results

Characteristics of the study population are summarized in Table 2. In total, 2395 cycles (TD: 998, CP-I: 747, CP-II-III: 650) were analyzed with ShTh and Sh, and 2099 cycles (TD: 934, CP-I: 681, CP-II-III: 484) with Feet. The corresponding cycles were selected for the reference system. No false positive gait events were detected. The number of non-detected cycles is shown in Table 3. Feet missed five cycles and ShTh/Sh missed three cycles for the TD group. Feet missed 197 cycles and ShTh/Sh missed 58 cycles for the CP group. Feet did not detect any events for one participant who walked exclusively on his toes.

**Figure 2:**
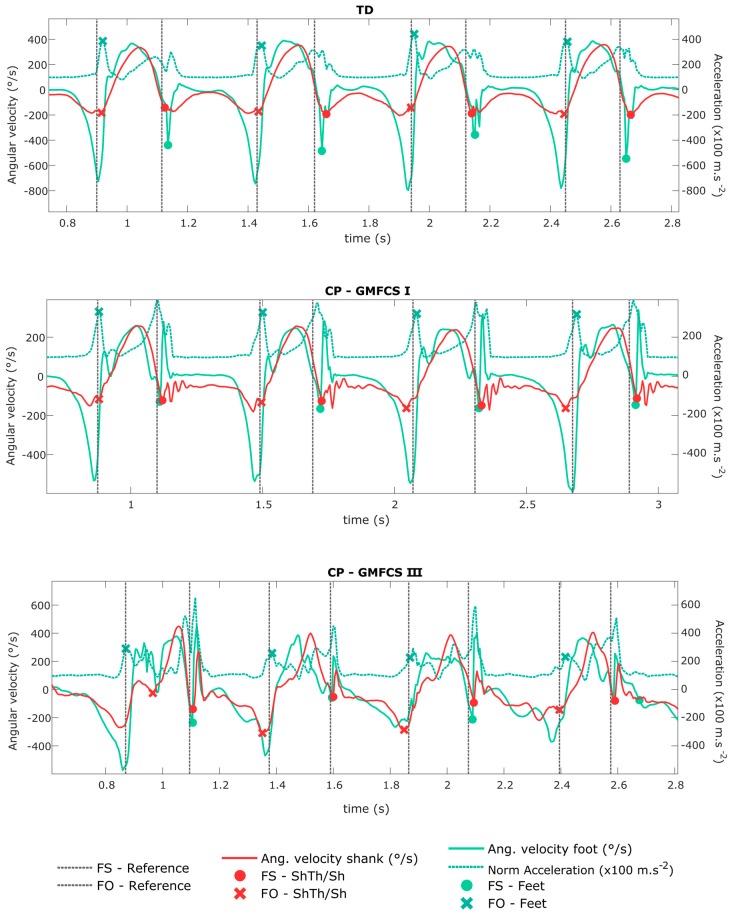
Shank angular velocity and foot angular velocity and norm of acceleration signals (measured by the wearable configurations ShTh/Sh and Feet). The gait events (FS and FO) detected by the wearable configurations are shown by the symbols on the corresponding signals. The vertical lines define FS and FO detected by the reference. Four strides are represented for each example, corresponding to a typically developing (TD) child, a child with CP-GMFCS I, and a child with CP-GMFCS III.

Table 3 summarizes the errors in estimating FS and FO times from the three configurations against the reference. Figure 2 illustrates an example for each group of FS and FO detection with ShTh/Sh and Feet configurations as compared to the reference. The three configurations showed similar errors for FS detection within the TD and the non-paretic groups (errors < 0.042 ± 0.030 s). Feet showed a higher error in detecting FS for the paretic groups (0.051 ± 0.053 s for GMFCS I and 0.077 ± 0.299 s for GMFCS II-III) compared to ShTh/Sh (0.037 ± 0.051 s for GMFCS I and 0.053 ± 0.048 s for GMFCS II-III). The error of FO detection was lower compared to FS detection, for the three configurations and the whole population (|errors| < 0.029 ± 0.058 s for FO and |errors| < 0.077 ± 0.299 s for FS).

Table 3 also reports the errors for STP computations (stride time, stride length, and stride velocity). The error of all configurations was inferior to 0.003 ± 0.072 s for stride time estimation within each group, except for the CP-GMFCS II-III group with Feet (0.012 ± 0.129 s). Feet showed lower errors compared to the two other configurations for stride length estimation, for all the groups except the CP-GMFCS I paretic group where the errors were equivalent between ShTh and Feet (0.019 ± 0.066 m and 0.018 ± 0.069 m). The highest errors for stride length estimation were found with Sh in each group. For stride velocity, ShTh showed lower errors for the CP groups (e.g., 0.024 ± 0.118 m/s (ShTh) against −0.127 ± 0.133 m/s (Sh) and 0.073 ± 0.123 m/s (Feet) in the GMFCS II-III group), whereas lower errors were found with Feet for the TD group (0.030 ± 0.045 m/s (Feet) against 0.073 ± 0.067 m/s (ShTh) and 0.133 ± 0.091 m/s (Sh)).

**Figure 3:**
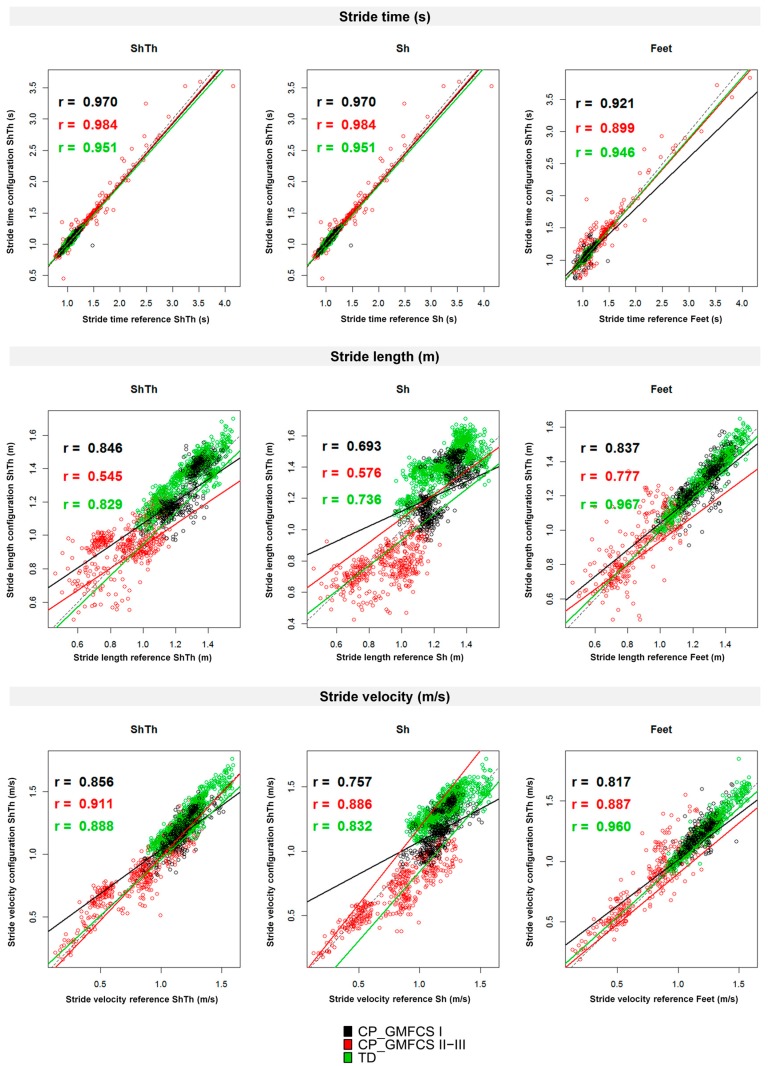
Correlations between the wearable configurations (ShTh, Sh, and Feet) and the optoelectronic system for the three spatiotemporal parameters (stride time, stride length, and stride velocity). Spearman’s correlation coefficients are reported. Dashed lines represent *y* = *x*. Green: TD; Black: CP-GMFCS I level; and Red: CP-GMFCS II-III levels.

Figure 3 presents the cycle-by-cycle comparison between each configuration and the reference for the three STP and the different groups. All correlations were significant (*p* < 0.05). Correlation coefficients were high to very high (*r* > 0.7) according to previous recommendations [45] for the three configurations except for stride length estimation in the CP-GMFCS II-III group with ShTh and Sh where the correlation coefficients were moderate (*r* = 0.545 and *r* = 0.576 respectively).

**Figure 4:**
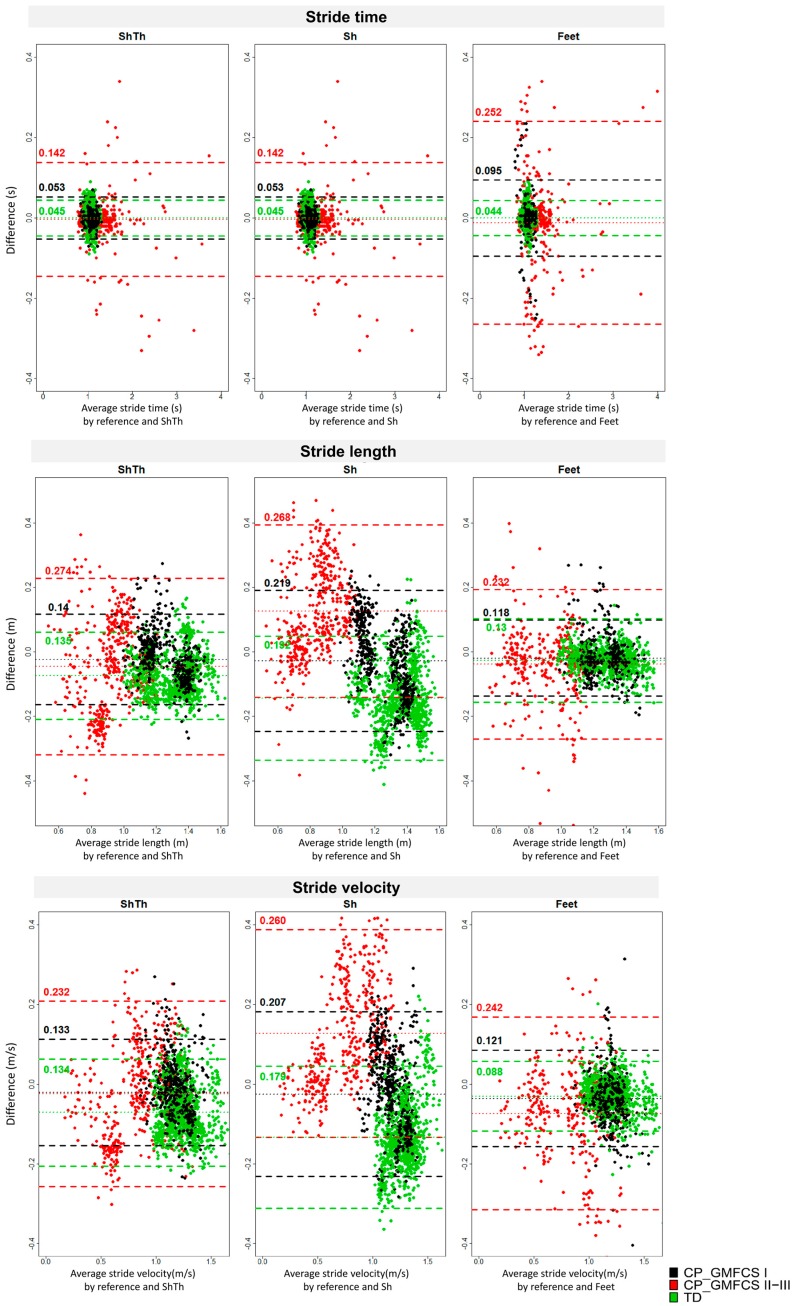
Bland–Altman plots of the three wearable configurations (ShTh, Sh, and Feet) against the optoelectronic system for the three spatiotemporal parameters (stride time, stride length, and stride velocity). Mean error and level of agreement (±1.96 standard deviation) are represented by horizontal lines. Green: TD; Black: CP-GMFCS I level; and Red: CP-GMFCS II-III levels.

The agreement assessed by Bland–Altman plots is shown in Figure 4. Higher levels of agreement were found for ShTh/Sh compared to Feet for stride time detection in CP-GMFCS I (0.095 s against 0.053 s) and CP-GMFCS II-III (0.252 s against 0.142 s). However, a higher agreement was reported with Feet for stride length in all groups as compared to the two other configurations. For the stride velocity, a better agreement was found with Feet for the TD and the CP-GMFCS I groups (0.088 m and 0.121 m/s) as compared to ShTh (0.134 m 0.0.133 m/s) and Sh (0.179 m and 0.207 m/s); however, ShTh showed a better agreement for CP-GMFCS II-III (0.232 m/s) as compared to Feet (0.242 m/s) and Sh (0.260 m/s).

## 4. Discussion

In this study, we aimed to compare three wearable configurations defined by different sensor positioning and associated algorithms in order to choose the most appropriate one for gait performance evaluation in children with CP. The main findings were that the configuration using sensors only on the shanks was not appropriate for the spatial and spatio-temporal estimation for the whole study population, and ShTh and Feet provided advantages depending on the estimated parameter and the level of severity of the child. Indeed, temporal parameters were better estimated by the ShTh configuration in the pathological groups, whereas spatial parameters were better estimated by the Feet configuration. For velocity estimation, the Feet configuration was better in TD children and children with a mild gait impairment (GMFCS I), but the ShTh configuration was better in children with more impaired gait (GMFCS II and III).

### 4.1. Temporal Detection

The key starting point of gait analysis is the segmentation of gait episodes into gait cycles using specific temporal events: FS and FO. For the whole study population, the three configurations were comparable regarding the detection of these events, with ShTh and Sh being more robust than Feet to detect events in challenging gait patterns (less non-detected cycles and better FS detection in the CP group compared to Feet).

When discussing the temporal results, one has to consider that the resolution frame of both systems was 0.01 s (sample frequency: 100 Hz), that the reference system error can reach 0.02 s [33], and that the systems’ synchronization precision was up to 0.01 s due to the delay between the trigger signal recorded by the additional Physilog and the actual start of the cameras. Considering these statements, FO detection with wearable systems was very close to FO detection of the reference (1 to 3 frames difference). For FS detection, the difference between the systems was larger (superior to 3 frames) especially for Feet for the most severely affected patients (up to 7 frames difference), indicating that ShTh/Sh was more accurate to segment gait trials and therefore appropriate to estimate the stride time for these patients. The errors of FS time related to the ShTh method in TD children was five times higher than those of the original method found by Salarian et al. including Parkinson’s disease and healthy adults [39]. This could be due to the difference in reference event detection or sampling frequency of the inertial sensors twice higher than in the present study.

The difficulty to detect events with inertial sensors is directly linked to, first, the pattern of the signal and, second, its smoothness. As illustrated in Figure 2, in abnormal gait patterns, expected features of the signal (such as specific thresholds, time windows, or peaks) can be missing, and non-expected features (such as extra-peaks) can appear which decrease the algorithm performance for event detection. In CP, these abnormal patterns are found especially at the distal parts of the limbs (at feet level) since movements of the distal limbs are more affected by the pathology than the proximal segments [46]. This explains why ShTh seems preferable for children with a high level of disability for gait events detection.

### 4.2. Spatial Detection

The Sh configuration was not suitable for children. Sh errors were between 3.5 and 3.8 times higher in our study compared to previously published results for healthy adults [40]. Indeed, with Sh, we found a systematic error for TD (11% of mean stride length) and CP-GMFCS II-III groups (14% of mean stride length). The Sh method uses a thigh predictor to estimate the thigh angles for stride length computation which has been trained on adults data (healthy and Parkinson’s disease patients). Even if the method aimed to suit any target population [40], it proved unfit for the children population, even the TD children. Furthermore, as the thigh angle is predicted from the shank sensor, misalignment of the sensor axis with the mediolateral axis of the shank has a greater impact on Sh results compared to the results where both thigh and shank angles are estimated from the thigh- and shank-fixed sensors. A calibration procedure that aligns the sensor frame with the anatomical frame of the shank would probably increase the performance of the system [40]. Such a calibration was not performed in this study as it was not straightforward for the heterogeneous and highly affected children we evaluated. The focus of this paper was the evaluation of errors before any calibration.

Previous studies found that STP computation with inertial sensors in healthy populations was improved when the sensors were closer to the ground [47,48] (i.e., closer to the measure). The results of this study partially confirmed this since Feet was found more accurate in average to estimate stride length compared to ShTh. On the other hand, Feet configuration was not able to analyze one toe-walking participant. Indeed, the Feet method is based on the assumption that the participant achieves ‘foot-flat’ contact at mid-stance to set the initial orientation of the sensor relative to the fixed frame [41]. This condition was not fulfilled for all children with CP included in this study, especially in the GMFCS II-III group, which caused the decreased performance of the algorithm. Overall, Feet configuration might be more accurate for STP parameters but is limited for highly abnormal gait patterns, which are more accurately estimated with sensors on shanks and thighs.

Finally, both ShTh and Feet configurations reported errors lower than the in-laboratory intra-subject variability of gait in children [49], so they can both be considered as valid and suitable for gait assessments.

### 4.3. Choice for Daily Life Assessment

The main perspective of this study is to determine the optimal configuration of sensors for the assessment of gait in the daily-life of children with CP. While measurement errors were the main criteria of comparison between the three systems, other aspects require careful consideration when choosing the best set-up for daily-life application.

On one hand, it is useful to keep the number of sensor units as low as possible to increase comfort for the patient, decrease the set-up time, and thus, make the system acceptable for long measurements [50]. Feet sensors were embedded in a droplet fixed directly on the skin to be independent of shoe wearing in order to allow the monitoring of all gait episodes within a day, including various environments. However, this system sometimes provided discomfort when tested outside the laboratory, because the shoe pressuring on the sensor droplet caused pain. Even if the shank and thigh sensors were larger, they did not hinder the children’s gait and were more convenient. However, the positioning of sensors on the shanks might need to be adapted for the children while wearing orthoses. On the other hand, more sensors can detect a higher variety of gait parameters and activities which confer a substantial advantage for daily life assessment [51,52]. ShTh configuration allows the recognition of the sitting, lying, and standing posture whereas Feet configuration cannot distinguish sitting from standing posture. Computation of complementary parameters, valuable for the assessment of children with CP, must also be considered. For example, foot clearance and foot/floor angles can be computed only with the Feet configuration, and knee angle only with the ShTh method.

Combining these practical aspects with the accuracy of the measurements, we selected the configuration using shank and thigh sensors for further testing of its usability for long-term assessments in laboratory-free conditions.

### 4.4. Limitations and Perspectives

The results of this study were obtained in a controlled environment which is not representative of daily-life conditions. Consequently, in daily-life settings, environmental disruptions, incorrect set-up, unintentional switch-off, or sensor fall might downgrade the results. Our laboratory setting reference, even though considered a ‘gold standard’, can reach 0.02 s in timing error [33] and a mean accuracy of 5.3 mm for marker trajectory estimates [9]. The errors reported can, therefore, be a combination of errors of both systems. Furthermore, only configurations using sensors on the lower limbs have been tested in this study. Other configurations including those using sensors on the wrists, the chest, or the pelvis exist [21,53] but were not tested as we wished to analyze the paretic and non-paretic limbs separately. Finally, we evaluated a limited number of subjects and, since CP gait patterns are very heterogeneous, a larger number of participants, especially in the CP group with a high level of disability (GMFCS II-III), would permit to strengthen the conclusions. Besides this, an increased number of participants would allow subgroup analysis within the GMFCS II-III group according to what algorithms could then be refined to be more versatile and flexible enough to fulfill the requirements of every individual clinical picture.

## 5. Conclusions

We compared three configurations of wearable sensors able to compute STP in children with CP and TD children on the base of the comparison with an optoelectronic system in the laboratory. The results showed that the configuration using sensors on both feet was more accurate for typical and regular gait patterns (i.e., for of TD children), while sensors located on the shanks and thighs performed better for moderate to severely impaired gait patterns (CP with GMFCS levels II and III). Overall, the results of this study indicate that inertial sensors proved promising for an objective evaluation of gait in daily-life. Such evaluations have the potential to shed light on patients’ daily difficulties.

## Figures and Tables

**Table 1:** Description of the three wearable configurations: ShTh, Sh, and Feet according to the sensors placement and associated algorithms.

Configuration Name		Shanks and Thighs	Shanks	Feet	
Abbreviation		ShTh	Sh	Feet	
**Sensor placement**		On the anterior side of each shank and thigh	On the anterior side of each shank	On top of each foot	
**Associated algorithm**	**Authors**	Salarian et al. 2004 [39]	Salarian et al. 2013 [40]	Mariani et al. 2010, 2013 [41,42]	
	**Calibration on sensor axis**	No calibration needed as the pitch axis is assumed systematically aligned with the mediolateral axis of the body		Sensor axes vertically aligned using the gravity component during motionless periods and the orientation was obtained by maximizing the pitch angular velocity of the foot throughout the gait trial.	
	**Events (temporal) detection method**	Peak detection on pitch angular velocity of shanks		Peak detection on pitch angular velocity and norm of acceleration of feet	
	**Spatial detection method**	Application of a double pendulum model [20] from shank and thigh angles (calculated by integration of shanks and thigh angular velocities) and segments lengths.	Application of a double pendulum model [20] from shank and thigh angles estimated only from shanks angular velocities and segments lengths. Thigh angles estimated from shank angles using Fourier series and least square optimization.	De-drifted double integration of gravity-free acceleration between two successive and identical events (foot flat)	
	**Spatio-temporal computation**	Temporal parameter divided by spatial parameter		De-drifted single integration of gravity-free acceleration between two successive and identical events (foot flat)	
	**Outputs gait parameters**	*n* = 24; includes: - Gait event times (foot strike (FS), foot off (FO)) - Temporal parameters (cadence, stride time, swing time, stance time, …) - Stride length, stride velocity - Shank, thigh and knee angle range of motion - Maximal peak angular velocity of shank		*n* = 55; includes: - Gait event times (FS, FO) - Temporal parameters (cadence, stride time, swing time, stance time, …) - Stride length, stride velocity - Turning angle - Foot clearances features, foot angles at FS, FO	
	**Population used for validation**	Healthy adults, elderly, patients with a total hip replacement, patients with coxarthrosis [20,43], and Parkinson’s disease (PD) adults [39]	Healthy adults, patients with a total hip replacement, patients with coxarthrosis, and PD patients [40].	Young, elderly adults and PD patients [41,44]	Children with cerebral palsy (CP) [29]
	**Mean errors (±sd) against reference**	Against force plate and optoelectronic system (ELITE, BTS, Milan, Italy) - Stride time: 0.002 (±0.023) s - Stride length: 0.035 (±0.085) m - Stride velocity: 0.030 (±0.076) m/s Against Instrumented walkway (GaitRite, Franklin, USA): - Stride time: 0.013 (±0.020) s - Stride velocity: 0.04 (±0.07) m/s	Against force plate and optoelectronic system (ELITE, BTS, Milan, Italy) - Stride time: 0.002 (±0.023) s - Stride length: 0.038 (±0.066) m - Stride velocity: 0.038 (±0.056) m/s	Against force plate and optoelectronic system (Vicon, Oxford Metrics, Oxford, UK) - Stride length: 0.034 (±0.046) m - Stride velocity: 0.043 (±0.042) m/s For PD adults: - Stride length: 0.013 (±0.030) m - Stride velocity: 0.028 (±0.024) m/s	Against force plate and optoelectronic system (Vicon, Oxford Metrics, Oxford, UK) - Stride length: 0.040 (±0.052) m - Stride velocity: 0.051 (±0.048) m/s

**Table 2:** General characteristics of the study population.

	TD	CP	
**N**	11	15	
		**GMFCS I**	**GMFCS II-III**
		7	8
		Unilateral	Bilateral
		5	10
**Sex** Number of girls in the group (%)	5 (45)	8 (55)	
**Age in years** mean ± std	13.5 ± 2.9	12.8 ± 3.1	

**Table 3:** Number of non-detected cycles, mean values (and standard deviation), mean errors (and standard deviation) against the optoelectronic system for gait event (Foot strike and Foot off) detection and spatiotemporal parameters (stride time, stride length, and stride velocity) computation. Positive/negative values stand for late/early gait event detection respectively and over/underestimation of STP. Reference values are in bold.

		TD		CP					
				GMFCS I				GMFCS II-III	
		Healthy sides		Non-paretic side		Paretic side		Paretic sides	
**Cycle detection**		*n* (%)		*n* (%)		*n* (%)		*n* (%)	
Number of non-detected cycle	ShTh/Sh	3 (0.1)		0 (0.0)		0 (0)		58 (2.4)	
	Feet	18 (0.8)		24 (1.0)		3 (0.1)		170 (7.4)	
**Gait event**		Error mean (SD)		Error mean (SD)		Error mean (SD)		Error mean (SD)	
Foot strike	ShTh/Sh	0.040 (0.021)		0.042 (0.030)		0.037 (0.051)		0.053 (0.048)	
(s)	Feet	0.047 (0.020)		0.048 (0.020)		0.051 (0.053)		0.077 (0.299)	
Foot off	ShTh/Sh	−0.011 (0.021)		−0.004 (0.029)		−0.008 (0.045)		−0.011 (0.063)	
(s)	Feet	0.012 (0.021)		0.014 (0.018)		0.020 (0.018)		0.029 (0.058)	
**STP**		Mean (SD)	Error	Mean (SD)	Error	Mean (SD)	Error	Mean (SD)	Error
			mean (SD)		mean (SD)		mean (SD)		mean (SD)
Stride time	**Reference**	**1.061 (0.080)**		**1.095 (0.073)**		**1.055 (0.092)**		**1.197 (0.388)**	
(s)	ShTh/Sh	1.064 (0.082)	0.000 (0.023)	1.091 (0.090)	0.001 (0.017)	1.055 (0.091)	0.000 (0.031)	1.200 (0.393)	0.003 (0.072)
	Feet	1.061 (0.083)	0.000 (0.022)	1.096 (0.073)	0.001 (0.019)	1.052 (0.101)	0.000 (0.057)	1.312 (0.431)	0.012 (0.129)
Stride length	**Reference**	**1.276 (0.144)**		**1.267(0.108)**		**1.242 (0.104)**		**0.896 (0.161)**	
(m)	ShTh	1.351 (0.135)	0.075 (0.069)	1.297 (0.145)	0.030 (0.078)	1.262 (0.134)	0.019 (0.066)	0.942 (0.143)	0.046 (0.140)
	Sh	1.420 (0.133)	0.144 (0.098)	1.298 (0.179)	0.031 (0.124)	1.269 (0.157)	0.027 (0.104)	0.770 (0.118)	−0.126(0.137)
	Feet	1.295 (0.151)	0.025 (0.037)	1.296 (0.095)	0.023 (0.036)	1.257 (0.118)	0.018 (0.069)	0.916 (0.182)	0.039 (0.118)
Stride velocity	**Reference**	**1.201 (0.155)**		**1.167 (0.110)**		**1.184 (0.104)**		**0.810 (0.283)**	
(m/s)	ShTh	1.277 (0.154)	0.073 (0.067)	1.193 (0.129)	0.026 (0.072)	1.201 (0.129)	0.018 (0.065)	0.834 (0.252)	0.024 (0.118)
	Sh	1.338 (0.115)	0.133 (0.091)	1.193 (0.160)	0.025 (0.116)	1.208 (0.143)	0.024 (0.098)	0.683 (0.212)	−0.127 (0.133)
	Feet	1.233 (0.165)	0.030 (0.045)	1.200 (0.093)	0.036 (0.039)	1.216 (0.117)	0.035 (0.070)	0.831(0.286)	0.073 (0.123)
